# Perturbation of Intracellular Cholesterol and Fatty Acid Homeostasis During Flavivirus Infections

**DOI:** 10.3389/fimmu.2018.01276

**Published:** 2018-06-04

**Authors:** Joao Palma Pombo, Sumana Sanyal

**Affiliations:** ^1^HKU-Pasteur Research Pole, School of Public Health, The University of Hong Kong, Hong Kong, Hong Kong; ^2^School of Biomedical Sciences, Li Ka Shing Faculty of Medicine, The University of Hong Kong, Hong Kong, Hong Kong

**Keywords:** cholesterol, lipid metabolism, virus, innate immunity, fatty acid

## Abstract

Cellular lipid homeostasis is maintained through an intricately linked array of anabolic and catabolic pathways. Upon flavivirus infections, these are significantly altered: on the one hand, these viruses can co-opt lipid metabolic pathways to generate ATP to facilitate replication, or to synthesize membrane components to generate replication sites; on the other hand, more recent evidence suggests counter strategies employed by host cells, which actively modulate several of these networks in response to infection, enhancing interferon signaling by doing so, and thus creating an antiviral environment. In this review, we discuss recent data on mechanisms of alteration of lipid metabolic pathways during infection by flaviviruses, with a focus on cholesterol and fatty acid biosynthesis, which can be manipulated by the invading viruses to support replication, but can also be modulated by the host immune system itself, as a means to fight infection.

## Introduction

Metabolic reprogramming in immune cells is a recurrent phenomenon when exposed to pro-inflammatory stimulants in the form of pathogens or cytokines. Macrophages and dendritic cells in particular are well-equipped to sense and respond to impending danger by pathogens, thus establishing the frontline of host defenses. Recent studies have highlighted the extraordinary contribution that multiple host metabolic pathways confer toward the ability of innate immune cells to respond to infections (1). Not surprisingly, some of the very same pathways that function to eradicate infection are often rewired by the invading pathogen.

Most viruses are known to induce aerobic glycolysis akin to the Warburg effect (2, 3). More recently, perturbation in lipid metabolic pathways has also been reported for several classes of pathogens (4, 5). Intracellular lipid homeostasis is achieved through a balance in biosynthetic, transport, and degradation processes. Current evidence increasingly points toward an intricate relationship between host lipid metabolism and intracellular pathogens, including bacteria, viruses, and parasites. While the mechanistic details are yet to be unraveled, it is hypothesized that these pathogens, on account of their limited genome sizes, co-opt the host metabolic network to meet the energy demands and procure precursors for their anabolic processes including replication and intracellular transport. In addition, viruses alter lipid metabolism to facilitate amplification and evade the host immune response. This has been decidedly observed in cases of positive strand RNA virus infections, such as dengue, West Nile, Hepatitis C, and several coronaviruses (6–10). Marked alterations in cholesterol and fatty acid biosynthesis occur upon infection, accompanied by the appearance of distinctive compartments, believed to be their replication sites (11–14).

Despite diversity in their genome organization, many viruses share certain salient features, primary of which is their dependence on host factors to undergo replication, assembly, intracellular transport, and release (15–20). The intracellular life cycle of positive strand RNA viruses is largely confined to the cytosol, within or on the surface of virus-induced organelle-like structures regarded as replication compartments (21–26). Notwithstanding differences in transmission, host cell tropism, and pathogenesis, these viruses employ similar strategies for replication and assembly, often accompanied by reorganization of the host secretory pathway (13, 24, 25, 27, 28). The replication sites serve multiple purposes that function in a concerted fashion to facilitate efficient virus propagation. Primarily, they offer spatial segregation of the different steps in the intracellular life cycle, such as RNA translation, replication, and packaging of the viral genome into virions during assembly. Viral replication compartments also enable a high local concentration of the necessary components—both viral and host—in a physically constrained space, ensuring efficient RNA amplification. An equally important feature of these replication sites is to limit exposure of viral RNA to the hostile cytoplasmic environment that contains cellular nucleases and sensors of the innate immune surveillance. Degradation of dsRNA replication intermediates is minimized by protection in membrane-delimited compartments.

Although lipid metabolism has received particular attention with Gram-negative bacterial infection, several recent reports highlight their function in viral infections (29). Analogous to lipopolysaccharide (LPS)-mediated downregulation of sterol synthesis in case of viral infections, limiting cholesterol biosynthesis in human macrophages and fibroblasts *via* genetic knockdown of sterol regulatory element-binding proteins [sterol-regulatory element-binding proteins (SREBPs), discussed in a later section], was reported to spontaneously engage type I IFN signaling and restrict infection (30–33). Initiation of anti-viral immunity thus displays a clear link with intracellular cholesterol biosynthesis, in a way that the induction of cholesterol synthesis would allow subversion of host immune responses and facilitate viral multiplication.

With the advent of omics-based studies, it is increasingly becoming obvious that viruses induce large-scale alterations in host cellular metabolism (3, 34–37). Among other examples are the induction of fatty acid synthesis by hepatitis C virus (HCV) in human hepatocytes, and the utilization of cellular lipid stores of hepatocytes by dengue virus. The effects of these events have been experimentally demonstrated by genetic and pharmacological inhibition of lipid biosynthetic pathways that attenuate viral pathogenesis (5, 38). These viral adaptation strategies can effectively increase available energy for virus replication and assembly, provide specific components for progeny particles, and for creating replication sites while suppressing antiviral signaling cascades. These reports highlight the intricate link between viruses and lipid metabolism. In the following sections, we discuss emerging data on fatty acid and cholesterol biosynthetic pathways that are upregulated by certain viruses to facilitate infection.

## Upregulation of Cholesterol and Fatty Acid Syntheses During Virus Infections

### Fatty Acid Synthase (FASN)

Intracellular contents of fatty acids and cholesterol contribute to fuel storage as well as a source of components necessary for increased membrane production. The core reaction of fatty acid synthesis is catalyzed by FASN starting from acetyl CoA and malonyl CoA. Once synthesized, palmitate can have several different fates, including further elongation to long chain fatty acids, which can be used for membrane production or storage in lipid droplets (LDs) in the form of triacylglycerols and esterified cholesterol. LDs are storage organelles consisting of triacylglycerols and steryl esters, and function as inert storage depots of excess cellular lipids. Abundance and size of LDs could be indicative of increased fatty acid synthesis, which might poise the cell for rapid membrane generation if needed and also maintains energy reserves (39). According to cellular states and their corresponding energy demands, fatty acids undergo β-oxidation to generate acetyl CoA and NADH and FADH_2_ molecules in the mitochondrial matrix, for ATP production *via* oxidative phosphorylation. Viruses induce and require availability of fatty acids at several stages of their lifecycle—either to supplement energy requirements for their anabolic processes or to generate viral replication compartments, most notably observed during infection by positive strand RNA viruses (40, 41). This is primarily due to the process of replication—confined to the cytoplasm—where such viruses alter the host intracellular lipid composition to create a beneficial environment. This phenomenon is exemplified by HCV, where all aspects of the viral lifecycle, including entry, replication, assembly, and release are host lipid associated (8). HCV requires low density lipoprotein receptor as a co-factor for entry into target cells (42). Its replication occurs in membranous web-like compartments referred to as double membrane vesicles (13, 43) and they assemble using LDs as platforms (18, 44). To generate replication sites, HCV triggers synthesis of fatty acids, cholesterol, and LDs (45–48). Another member of the Flaviviridae family, dengue, has also been reported to induce production of fatty acids (49, 50). FASN and ACC1 were identified through a targeted siRNA screen as necessary factors for efficient dengue virus replication (38, 51). Drugs that inhibited FASN activity resulted in a significant attenuation in virus replication (49). Infection with dengue virus does not affect FASN expression levels, but rather its redistribution to virus-triggered structures referred to as convoluted membranes (50). This phenomenon appears to be Rab18-mediated, a member of the GTPase family that typically resides in the ER and LDs. Upon infection dengue NS3 was found to interact with Rab18, which allowed recruitment of FASN to viral replication sites, thus promoting fatty acid biosynthesis to increase their local concentration (51, 52). Inhibiting FASN activity has a similar effect in mosquito cells with loss of infectious progeny virion production (53).

### 3-Hydroxy-3-Methylglutaryl-CoA Reductase (HMGCR)

3-hydroxy-3-methylglutaryl-CoA reductase is the rate-limiting enzyme for cholesterol biosynthesis and is regulated *via* a negative feedback mechanism mediated by products of the mevalonate pathway. In mammalian cells, HMGCR activity is suppressed by cholesterol imported through receptor-mediated endocytosis of low density lipoproteins (54). Dengue infection inhibits phosphorylation of HMGCR at an inactivating site, generating a cholesterol-rich environment in the process (55, 56). This was further corroborated through inactivation of AMPK and a subsequent increase in HMGCR activity, respectively (56). In comparison, West Nile virus infection has a more direct impact on intracellular cholesterol distribution. Infection was accompanied by redirecting cholesterol from the plasma membrane to virus replication sites (12). In mammalian cells cholesterol homeostasis is tightly regulated in a feedback mechanism *via* transcription factors that sense intracellular cholesterol levels (57, 58). These transcription factors are termed SREBPs that associate tightly with the sterol-sensing SREBP cleavage-activating protein (SCAP) within the ER membrane, *via* an additional interaction with the ER-resident protein Insig, which functions as an inhibitor of SREBP (59, 60). SCAP has an additional role as a chaperone that mediates transport of the SREBP–SCAP complex to the Golgi network, where SREBP is proteolytically cleaved by two resident Golgi proteases (S1P and S2P) to release the transcriptionally active fragment of SREBP from the membrane. The released forms of SREBPs are transported to the nucleus and activate transcription of target genes required for cholesterol and fatty acid biosynthesis, including HMGCR and FASN, respectively. When cholesterol levels are high, SCAP binds to cholesterol in the ER, promoting an association with Insig, and retains the complex within the ER, thus reducing the synthesis of cholesterol. Conversely, when cholesterol levels are low, binding of SCAP to Insig is disrupted, and cholesterol synthesis is initiated (61–63). The authors of these studies postulated that de-enrichment of cholesterol from sites harboring sensory molecules, such as the SCAP–SREBP–Insig complex, results in activation of this signaling pathway, enabling the host cell to increase cholesterol levels to accommodate proliferation of intracellular membranes.

## Modulation of LDs During Virus Infections

**Figure 1:**
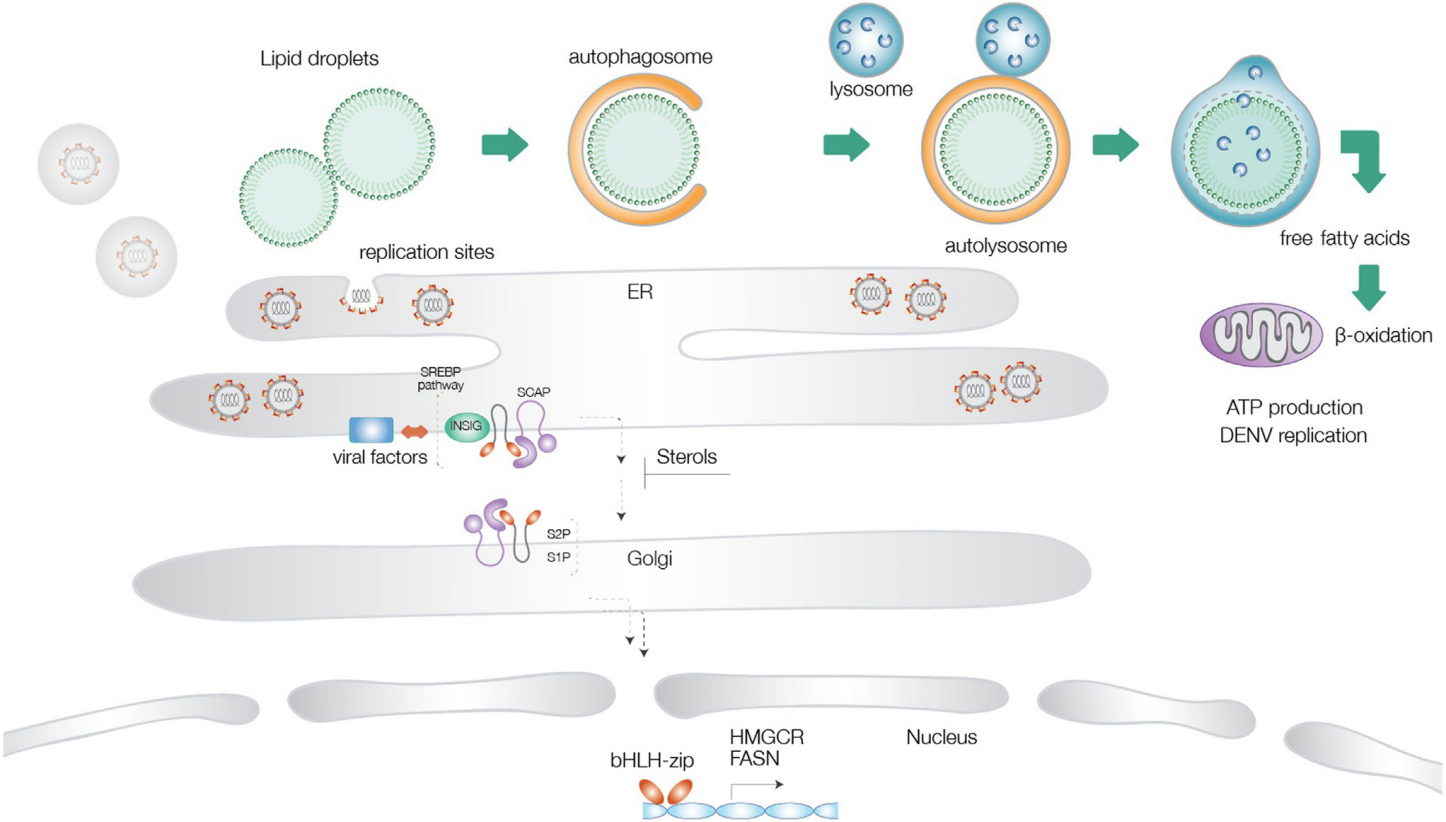
Schematic illustration of the different lipid metabolic pathways exploited during flavivirus infections. Virus-triggered activation of lipophagy results in generation of cholesterol and free fatty acids, which undergo β-oxidation in the mitochondrial matrix to increase cellular energy levels. Activation of the sterol-regulatory element-binding proteins (SREBP) pathway at the endoplasmic reticulum is followed by transport of the SREBP cleavage-activating protein-SREBP complex to the Golgi network, proteolysis, and translocation of the transcription factor to the nucleus, where it turns on genes for cholesterol and fatty acid biosynthesis. Several flaviviral factors have been implicated in exploitation of this pathway. Increased FA production provides energy supplies necessary for virus replication and components necessary for generation of replication sites.

Lipid droplets are multifunctional organelles present in most organisms from bacteria to eukaryotes (64–66). These structures are particularly abundant in mammalian adipocytes and insect fat body cells. LDs are mainly composed of a phospholipid monolayer and structural proteins, such as Perilipins, which are involved in LD biogenesis and degradation. Despite previous notions on a rather static role of LDs in the maintenance of lipid homeostasis, more recently, it has become evident that LDs are also present in immune cells, such as neutrophils and macrophages, where they regulate inflammatory or infectious processes (65, 67). Upon stimulation with different challenges, they display an increase in abundance and thereby serve as reliable markers of immune cell activation. Autophagy dependent degradation of LDs has been reported for dengue virus infection in human hepatocytes (38). A similar activation of the autophagy pathway was recently described for Zika virus infection as well (68). Our own data (accepted, queued for publication) support a drastic upregulation of LD consumption through induction of autophagy upon both dengue and Zika virus infections. This pathway appears to operate in an ancient ubiquitous protein 1 (Aup1)-dependent manner, and is dictated by its ubiquitylation status. Unmodified Aup1 enabled dispersion of LDs, which underwent lipophagy upon infection. This virus-triggered pathway is essential for assembly and production of newly synthesized progeny virions (in press). Current consensus, therefore, supports a model where mobilization of LDs in combination with increased synthesis of fatty acids and cholesterol provides a proviral environment for production of progeny virions (53) (Figure 1).

## Reconfiguring Cholesterol Metabolism as Host Response to Infection

The interdependence of innate immune signaling processes and the regulation of sterols and fatty acid metabolism is increasingly being consolidated through emerging data (30). Their role in production of inflammatory mediators has been reported by several groups (69–71). Interferons (IFNs) modulate the expression of a multitude of IFN-stimulated genes including viperin, which has been observed to be highly upregulated in response to bacterial LPS, double-stranded DNA, and RNA analogs, and also possesses antiviral activity against a range of viruses including HCV and dengue virus (72). In a similar vein, inhibition of cholesterol biosynthesis also exerts an antiviral effect (12, 73, 74). SREBPs are involved in coordinating the regulation of the sterol and fatty acid biosynthesis pathways; IFNs effectively inhibit SREBP2 at both mRNA and protein levels. Interestingly, WNV-induced redistribution of cellular cholesterol was found to downregulate IFN-stimulated JAK–STAT antiviral signaling response to infection, potentially by removing cholesterol from their usual microenvironment.

Recent evidence suggests that alterations to cellular lipid metabolism have a more direct role in host defense, through positive regulation of the type I IFN-mediated antiviral response: for example, activation of type I IFN signaling can induce upregulation of β-oxidation and inhibition of cholesterol synthesis, in order to create a hostile cellular environment for viruses (31, 75). Intracellular pathogens are known to stimulate *de novo* lipid and cholesterol biosynthesis to ensure their own survival. Accordingly, repressing these anabolic pathways can inhibit the evolution of intracellular infections. Activation of type I interferon receptors has been correlated to inhibition of cholesterol biosynthesis; however, repression of lipid metabolism in this manner is accompanied by an increase in the influx of environmental lipids, which maintain intracellular lipids and cholesterol at normal levels. Thus, type I IFN signals reprogram cellular lipid metabolism, but this does not function to limit lipid availability to pathogens. It, therefore, remains unclear whether IFN-I linked repression of cholesterol biosynthesis, in the context of intracellular infection, is meant to limit nutrient availability to pathogens, or if it serves a different purpose.

### Type I Interferon Response and Lipid Homeostasis During Infection

Bone marrow-derived macrophages (BMDMs), when challenged with IFN-β, poly:IC, or viral infection, showed decreased intracellular synthesis of fatty acids and cholesterol, as well as increased uptake of extracellular lipids and cholesterol. This was also demonstrated by a lower expression of genes related to cholesterol and fatty acid metabolism and an enhanced expression of genes related to cholesterol and lipid import, post viral challenge. Suppressing interferon alpha/beta signaling, while infecting BMDMs with virus, nullified all changes in lipid and cholesterol intracellular balance, including the gene expression level, which proves that type I IFNs can shift lipid homeostasis from biosynthesis to import, despite not significantly altering the intracellular levels of cholesterol and fatty acids (31).

### Crosstalk Between Lipid Metabolism and the Type I Interferon Pathway

The SCAP protein acts as a sterol-sensing element, as well as a chaperone, which associates with immature SREBP transcription factors in the ER membrane. By knocking out or knocking down SCAP in macrophages, SREBP activity is lowered, as well as expression of genes involved in lipid metabolism. As anticipated, *de novo* synthesis of cholesterol and fatty acids went down in the absence of SCAP, but total intracellular lipid levels remained unchanged. Loss of SCAP also correlated with heightened resistance to viral infection in *in vitro* and *in vivo* models, confirming the functional equivalence between activation of type I interferon pathway and inhibition of lipid metabolism. Culture medium supernatants from SCAP^−/−^ macrophage cultures were enough to markedly increase resistance to viral challenge, when supplied to wild-type BMDMs, suggesting that the higher type I interferon-mediated viral resistance was a causal effect of a secreted effector molecule, such as interferon-beta (IFNβ). In light of this, qPCR analysis revealed that both SCAP^−/−^ BMDMs and alveolar macrophages extracted from SCAP^−/−^ mice constitutively express higher levels of IFNβ and interferon-stimulated genes (ISGs), compared to wild-type macrophages. Finally, blocking the interferon alpha/beta receptor (IFNAR) was enough to restore interferon and ISGs expression back to normal levels, as well as losing resistance to viral infection. These data strongly suggested that the absence of SCAP activity spontaneously triggers type I interferon production, which translates into a constitutive state of higher resistance to viral infection in macrophages (31).

### Deficiency in Cholesterol Metabolism Triggers Type I Interferon Response

SREBP1 generally drives transcription of genes related to fatty acid metabolism, whereas SREBP2 activates transcription of genes linked to cholesterol biosynthesis. RNA-seq and qPCR analysis revealed that knockdown of SREBP1 in macrophages did not significantly impact IFNβ or ISG expression, whereas knockdown of SREBP2 caused a distinct increase in expression of IFNβ and several ISGs, not only in immune cells (macrophages) but also in non-immune cells (fibroblasts). Resistance to viral challenge was highly increased in SREBP2-deficient macrophages and SREBP2^−/−^ mouse fibroblasts (31). Moreover, blocking IFNAR in SREBP2-deficient cells restored ISGs to normal expression levels, decreasing resistance to virus infection to wild-type levels as well. This suggests that a higher type I interferon response is specifically caused by an inhibition of cholesterol metabolism (31, 32). In support of this hypothesis, cells (immune and non-immune) with deficiency in the mevalonate pathway showed a constitutively exacerbated type I interferon response. Also, addition of free cholesterol to SREBP2-deficient cells, or to cells with genetically impaired cholesterol metabolism, causes the exaggerated type I interferon response to decrease to basal levels (31, 71).

Stimulator of interferon genes (STING) is an ER resident kinase, which activates interferon regulatory factor 3 (IRF3) through phosphorylation of Tank binding kinase-1 (TBK1) (76). STING kinase activity is stimulated by cyclic dinucleotides, which are synthesized by cyclic GMP-AMP synthase (cGAS). cGAS, STING, and phosphorylated TBK1 (pTBK1) exist in higher basal levels in SREBP2^−/−^ cells compared to wild-type cells; in addition, knocking down either cGAS or STING in enough to drastically lower pTBK1 presence in SREBP2-deficient cells. Also, knockdown of cGAS, STING, or TBK1 in SREBP2^−/−^ cells caused the expression of Ifnb1 and ISGs to decrease to levels similar to those in wild-type cells (31).

Addition of free cholesterol to SREBP2-knockout cells significantly decreased pTBK1, while blocking IFNAR had no effect on pTBK1 levels, reinforcing the idea that cholesterol directly influences STING-mediated activation of TBK1. These data support a model in which a lack of cholesterol in the cell makes STING more sensitive to cyclic dinucleotides, upregulating the STING-pTBK1-IRF3 signaling axis, and ultimately increasing expression of Ifnb1 and ISGs, conferring an intrinsic pro-inflammatory phenotype to cholesterol-deficient cells (77). Admittedly, most of these experiments used MHV68; however, these conclusions may very well be relevant in other virus infections.

## Targeting Fatty Acid and Cholesterol Metabolism as an Antiviral Strategy

Repressing the cholesterol biosynthetic pathway through inhibitors of HMGCR is a common treatment for cardiovascular diseases (78). The clinical success of these inhibitors for human disorders provides strong support that targeting lipid metabolism can effective for human therapy. Elucidating the specific alterations incurred upon virus infections would allow novel therapeutic approaches to emerge through targeted inhibition of such metabolic pathways. IFNs or viral infections often result in induction of 25-hydroxycholesterol in macrophages—an antiviral effector, which broadly inhibits many enveloped viruses by interfering with membrane fusion (79). Whether it has an additional impact on activating the interferon signaling pathway is to be seen in future studies. Different strategies can be employed to interfere with virus infection, including those involving lipid utilization; notwithstanding, it is tempting to speculate that drugs already in clinical use against cholesterol and fatty acid metabolic pathways might be repurposed to boost antiviral immunity and provide resistance to infection.

## Author Contributions

JP and SS discussed and wrote the manuscript.

## Conflict of Interest Statement

The authors declare that the research was conducted in the absence of any commercial or financial relationships that could be construed as a potential conflict of interest.
